# Human Immunodeficiency Virus-1 Diversity in the Moscow Region, Russia: Phylodynamics of the Most Common Subtypes

**DOI:** 10.3389/fmicb.2019.00320

**Published:** 2019-02-26

**Authors:** Aleksey Lebedev, Natalya Lebedeva, Fedor Moskaleychik, Alexander Pronin, Elena Kazennova, Marina Bobkova

**Affiliations:** ^1^Laboratory of T-Lymphotropic Viruses, N.F. Gamaleya National Research Center of Epidemiology and Microbiology, Moscow, Russia; ^2^Moscow Regional AIDS Centre, Moscow, Russia

**Keywords:** HIV-1 subtypes, diversity, phylodynamics, epidemic, TMRCA, transmission route, Moscow region, Russia

## Abstract

This study analyzes the HIV-1 subtype diversity and its phylodynamics in Moscow region, which is the most densely populated area of Russia characterized by high rates of internal and external migration. The demographic and viral data from 896 HIV-infected individuals collected during 2011–2016 were analyzed. The study revealed broad diversity in the HIV-1 subtypes found in Moscow, which included A6 (85.1%), B (7.6%), CRF02_AG (1.2%) and URF_A6/B recombinants (4.2%). Other HIV-1 subtypes were detected as single cases. While A6 was most prevalent (>86.0%) among heterosexuals, injecting drug users and cases of mother-to-child transmission of HIV, subtype B (76.3%) was more common in men who have sex with men. Phylogenetic reconstruction revealed that the A6 sequences were introduced into the epidemic cluster that arose approximately around 1998. Within the subtype B, six major epidemic clusters were identified, each of which contained strains associated with only one or two dominant transmission routes. The date of origin of these clusters varied between 1980 and 1993, indicating that the HIV-1 B epidemic began much earlier than the HIV-1 A6 epidemic. Reconstruction of the demographic history of subtypes A6 and B identified at least two epidemic growth phases, which included an initial phase of exponential growth followed by a decline in the mid/late 2010s. Thus, our results indicate an increase in HIV-1 genetic diversity in Moscow region. They also help in understanding the HIV-1 temporal dynamics as well as the genetic relationships between its circulating strains.

## Introduction

HIV-1 is a rapidly evolving human pathogen, characterized by significant genetic diversity. The pandemic group M is subdivided into nine subtypes (A–D, F–H, J, K), each of which have been further divided into sub-subtypes (A1-A4, A6, F1-F2), and multiple inter-subtype recombinants (CRFs) (Foley et al., 2016). The global distribution of HIV subtypes is heterogeneous and varies significantly even within the same continent/country (Hemelaar et al., 2011; Bobkova, 2013).

The HIV epidemic started in Russia in the mid-1990s with an outbreak of the subtype A infection among intravenous drug users (IDUs) (Bobkov et al., 1998). As per the current nomenclature, the viral variant that caused the first epidemic and accounted for up to 90% of the infections is the sub-subtype A6 (formerly FSU-A or IDU-A) (Foley et al., 2016). During the following years, the HIV epidemic spread further affecting heterosexuals (HSX) and men who have sex with men (MSM). Ten years later sexual transmission accounted for about 37.3% of the new cases (FedAC, 2010).

With a shift in the dominant transmission route in Russia, the ratio of HIV subtypes also changed. The overall proportion of the non-A6-subtype increased from 7.0% in 2000 to 10.0–20.0% in 2010 (Bobkov et al., 1998; Thomson et al., 2009; Gashnikova et al., 2011). In the mid-2010s the prevalence of HIV-1 subtypes in Russia was as follows: A6 (~70.0%), B (~10.0%) and CRF063_02A1 (~7.0%) (Lapovok et al., 2017). The subtype B was predominant (82%) among MSM (Dukhovlinova et al., 2015; Kazennova et al., 2017). The sub-subtype A6 was dominant in all territories of Russia, except the Siberian region and bordering Central Asia, where CRF063_02A1 and CRF02_AG recombinant variants were highly prevalent (Lapovok et al., 2014; Gashnikova et al., 2017; Kostaki et al., 2018).

During the last few years, the recombinant CRF063_02A1 has been responsible for more than 71.0% of the new HIV infections among HSXs and IDUs in Central Siberia (Gashnikova et al., 2015). Such changes in the prevalence of HIV variants in Russia are likely to affect the state of infection in Moscow region, which is characterized by a high population density and high rates of internal and external migration. Moscow located in the European part of Russia is a highly developed transport hub, for communications both within and outside Russia. The first HIV infection in Moscow region (not Moscow city) was reported in 1994; by the end of 2018 the number of people living with HIV (PLHIV) in this area was estimated to be 41,949 and the prevalence among adults has reached 0.7%.

There have been few studies on HIV diversity in Moscow region. While sub-subtype A6 has been described as the causative agent of the first HIV outbreak among IDUs probably introduced from St. Petersburg (Bobkov et al., 2001; Diez-Fuertes et al., 2015), by 2010 no significant changes were reported in the spectrum of circulating HIV subtypes [A6 (93.4%) and B (6.6%)] in the region (Giliazova et al., 2010). However, to date, there has been no information regarding the evolutionary history and population dynamics of HIV in Moscow region.

In this study, we aim to analyze the HIV-1 genetic diversity and reconstruct the temporal dynamics of its most common subtypes in Moscow region, using phylogenetic and phylodynamic approaches.

## Materials and Methods

### Study Population

The study involved 896 patients who were diagnosed with HIV infection and received antiretroviral therapy (ART) between 2011 and 2016 at the Moscow RCAC which is 2.1% of PLHIV in this area. Three criteria for formation of study population have been applied: (1) well-documented history of HIV-infection in patients, (2) permanent residency in Moscow region, and (3) informed consent for the research study.

### Genotyping and Dataset

Plasma samples were genotyped using the ViroSeq HIV-1 Genotyping System (Abbott, USA). Multiple sequence alignments were made using MAFFT (Katoh et al., 2017)The total length of the alignment was 1299 nucleotides which covered the entire protease and partial reverse transcriptase (positions 2253–3551, HXB2-numbering). HIV- 1 subtyping was performed using the COMET HIV-1 (Struck et al., 2014) and REGA Subtyping Tool v3.0 (Pineda-Pena et al., 2013) and subsequently confirmed by phylogenetic analysis. New inter-subtype or inter-CRF sequences were analyzed using jpHMM (Schultz et al., 2012). Moscow region sequences for A6 (*N* = 5) and B (*N* = 9) (2007–2008 sampling years) from the Los Alamos HIV-1 database (https://www.hiv.lanl.gov) were additionally included in the analysis. The epidemiological clusters were identified in the initial dataset of A6 (*N* = 768) and B (*N* = 77) subtypes. The evolutionary population dynamics was estimated in the optimized dataset by excluding (1) presence of more than 1% of ambiguous bases, (2) sequences with especially high evolutionary rates and (3) potential late chronic infections by AIDS symptoms. Finally, 320 and 71 sequences for A6 and B subtypes, respectively were selected. All codons associated with major drug-resistance mutations (Johnson et al., 2011) were removed from the final sequence alignment.

### Phylogenetic Reconstruction and Phylodynamic Analysis

Selecting the best-fit model of nucleotide substitution was performed using jModelTest v.2.1.4 (Darriba et al., 2012). According to the Akaike information criterion (AIC), the best model for each dataset was General Time Reversible model with proportion of invariable sites and gamma-distributed rate variation among sites (GTR+I+G).The epidemiological clusters were identified using the maximum-likelihood phylogenetic analysis implemented by the IQ-TREE (Nguyen et al., 2015) with 1000 replicates for bootstrap and Shimodaira–Hasegawa (SH)-aLRT test. The clusters with an SH-aLRT support >0.9 were considered reliable.

The time of the most recent common ancestor (TMRCA) and the effective population size was estimated employing the Bayesian Markov Chain Monte Carlo (MCMC) approach using the BEAST v1.10.0 (Suchard et al., 2018). The temporal scale of the evolutionary process was inferred from the sampling dates of the sequences using a strict molecular clock model and the Bayesian Skyline coalescent tree prior. Comparing molecular clock models (as well as demographic models) in Bayesian framework were performed by calculating the AIC for MCMC with Tracer v1.7.1. Two independent MCMC chains were run of 50–500 × 10^6^ steps with logging every 2000 generations, excluding first 25%. Convergence of the chains was estimated based on the Effective Sample Size in Tracer v1.7.1. The parameter estimates with ESS > 200 were accepted. We summarized the maximum clade credibility trees from the posterior distribution of trees using Tree Annotator and visualized them in FigTree v1.4.0. The datasets were analyzed with TempEst (Rambaut et al., 2016) for evaluating the temporal signal sufficiency.

### Statistical Analysis

Statistical analysis was performed using STATISTICA v10.0 (StatSoft, USA) with discrete categorical data and the Pearson's chi-squared (or Fisher's extact) test. Differences were considered significant at *P* < 0.05.

## Results

### Study Population

We evaluated 896 HIV-1 infected patients with a median age of 35 years (range 1–67). The proportion of men and women was about the same (58.5 and 41.5%, respectively). The dominant transmission routes were heterosexual contact (49.8%), intravenous drug use (39.8%), mother-to-child transmission (MTCT, 5.5%) and MSM contact (4.2%). The demographic characteristics of patients grouped by subtypes are summarized in Table 1.

**Table 1:** General characteristics of HIV-1 infected patients from Moscow region classified on subtypes.

		**HIV-1 subtypes**					***P-*value^**b**^**
	**Total (*N* = 896, 100.0%)**	**A6 (*N* = 763, 85.1%)**	**B (*N* = 68, 7.6%)**	**URF_ A6/B (*N* = 38, 4.2%)**	**CRF02_AG (*N* = 11, 1.2%)**	**Others^**a**^ (*N* = 16, 1.8%)**	
Age (years)							0.883^c^
< 30	170 (19.0)	143 (84.1)	15 (8.8)	7 (4.1)	1 (0.6)	4 (2.4)	
30–35	332 (37.0)	289 (87.1)	21 (6.3)	13 (3.9)	5 (1.5)	4 (1.2)	
> 35	394 (44.0)	331 (84.0)	32 (8.1)	18 (4.6)	5 (1.3)	8 (2.0)	
Gender							<0.001^c^
Male	524 (58.5)	424 (80.9)	61 (11.6)	23 (4.4)	4 (0.8)	12 (2.3)	
Female	372 (41.5)	339 (91.1)	7 (1.9)	15 (4.0)	7 (1.9)	4 (1.1)	
Risk group							0.001^d^
HSX	446 (49.8)	384 (86.1)	28 (6.3)	17 (3.8)	8 (1.8)	9 (2.0)	
MSM	38 (4.2)	6 (15.8)	29 (76.3)	1 (2.6)	–	2 (5.3)	
IDUs	357 (39.8)	323 (90.5)	11 (3.1)	17 (4.8)	3 (0.8)	3 (0.8)	
MTCT	49 (5.5)	44 (89.8)	–	3 (6.1)	–	2 (4.1)	
Unknown	6 (0.7)	6 (100.0)	–	–	–	–	
Sampling years							0.542^d^
2011	101 (11.3)	91 (90.1)	4 (3.9)	3 (3.0)	1 (1.0)	2 (2.0)	
2012	139 (15.5)	117 (84.2)	11 (8.0)	7 (5.0)	2 (1.4)	2 (1.4)	
2013	102 (11.4)	87 (85.3)	8 (7.8)	4 (3.9)	–	3 (3.0)	
2014	185 (20.6)	160 (86.4)	15 (8.1)	4 (2.2)	2 (1.1)	4 (2.2)	
2015	232 (25.9)	192 (82.8)	18 (7.8)	14 (6.0)	4 (1.7)	4 (1.7)	
2016	137 (15.3)	116 (84.7)	12 (8.8)	6 (4.4)	2 (1.4)	1 (0.7)	

*HSX, heterosexuals; MSM, men who have sex with men; IDUs, intravenous drug users; MTCT, mother-to-child transmission*.

a *HIV subtype A1 (6.2%), C (6.2%), G (18.8%), F1 (6.2%), CRF01_AE (6.2%), CRF03_AB (12.6%), CRF63_02A1 (12.5%), and non-A6/B URFs (31.3%)*.

b *Value for the difference between HIV-1 subtypes; HIV-1 ≪others≫ subtypes were not considered in the analysis*.

c *Pearson's chi-squared test*.

d *Pearson's chi-squared test (Yates's correction)*.

### HIV Subtypes

Analysis of the pol sequences from HIV-1 infected patients confirmed the expected broad spectrum of viral subtypes in Moscow region (Table 1). These included 763 viruses (85.1%) classified as sub-subtype A6, 68 (7.6%) as subtype B, 11 (1.2%) as CRF02_AG and 16 (1.8%) as ≪other≫ subtypes. In addition to ≪pure≫ HIV-1 subtypes (or CRFs), 43 (4.8%) URFs containing genome segments of different subtypes were found. The sub-subtype A6 and CRF02_AG were found mainly in HSXs (50.3% and 72.7%, respectively) and IDUs (42.3 and 27.3%, respectively). Subtype B infected predominantly men (89.7%), through MSM (42.6%) and HSX (41.2%) exposures. Seven other HIV-1 subtypes were also identified.

**Figure 1:**
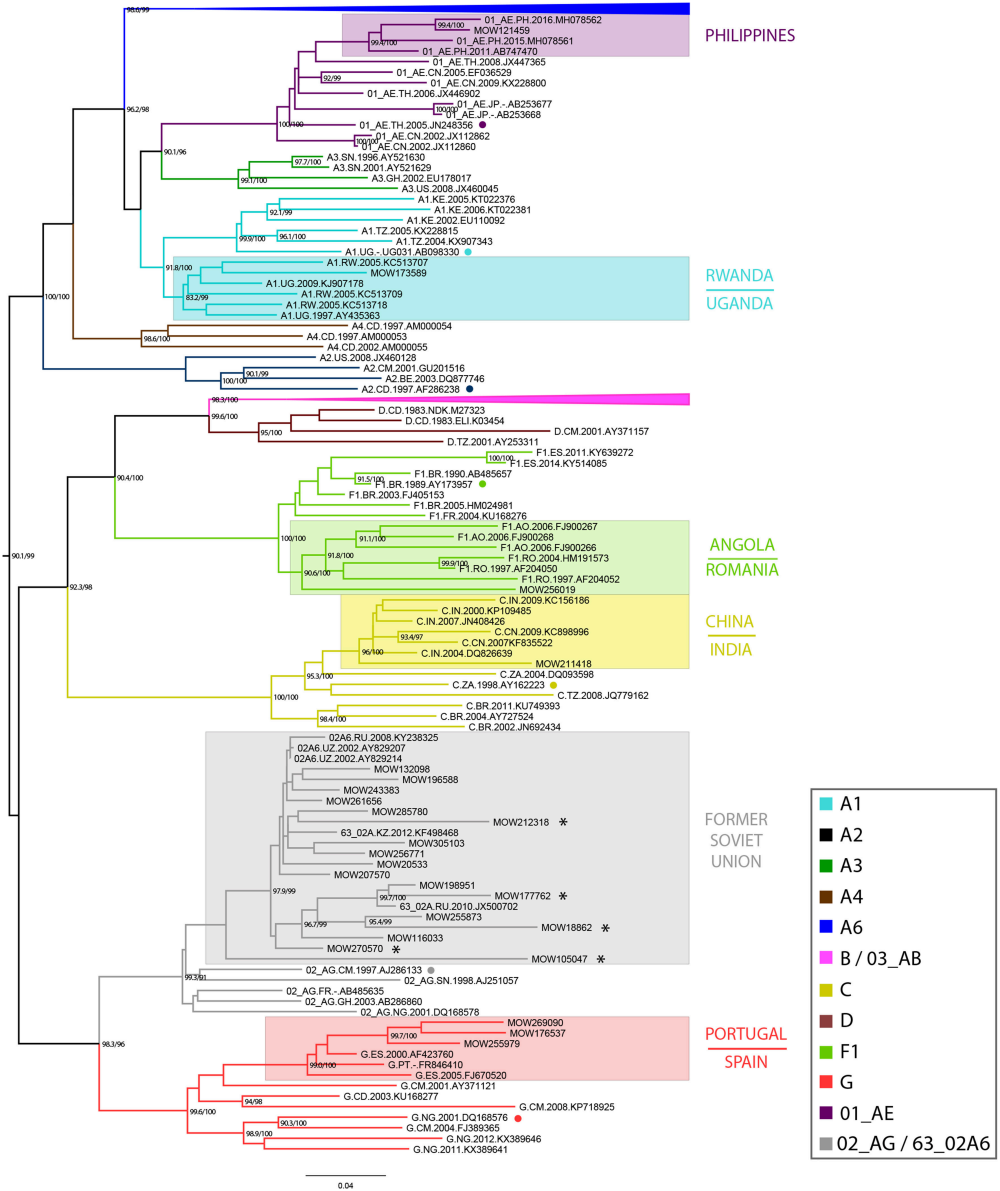
Maximum-likelihood phylogenetic tree of 896 HIV-1 pol sequences from HIV-infected individuals in Moscow region, from 2011 to 2016. The presented tree describes the analysis of all dataset, where the subtype A6 and B clades of HIV-1 are presented in a condensed form. The topology of the A6-clade and B-clade are presented separately (see Supplemental Figure 1). Branches are colored according to the HIV-1 subtypes as indicated in the legend. Support values (BS/SH-aLRT) are shown for the key nodes. Asterisks point to locations of unique recombination forms (URFs; see main text). The reference sequence for each subtype was selected using the Reference Alignment from Rega HIV-1 Subtyping Tool and marked by a circle.

Based on sequences identified by BLAST searches and phylogenetic analysis, sub-subtype A1 was found to be related to the East African strains (Uganda/Rwanda), subtype F1 to Angola and Romania, subtype C to the India/China C-strains; subtype G to the Portuguese/ Spanish G-strains and CRF01_AE to the Philippines AE-strains. Sub-subtype A6, CRF03_AB, CRF63_02A1, and CRF02_AG viruses were related to strains circulating in the former Soviet Union (FSU) countries. While most of the subtype B sequences (*N* = 64) belonged to the Pandemic (≪Western-B≫) variant, four sequences belonged to the FSU-B variant, previously identified among IDUs in Ukraine (Nabatov et al., 2002) (Figure 1).

### Identification of HIV-1 Subtypes A6 and B Epidemic Clusters, and TMRCA Estimation

**Figure 2:**
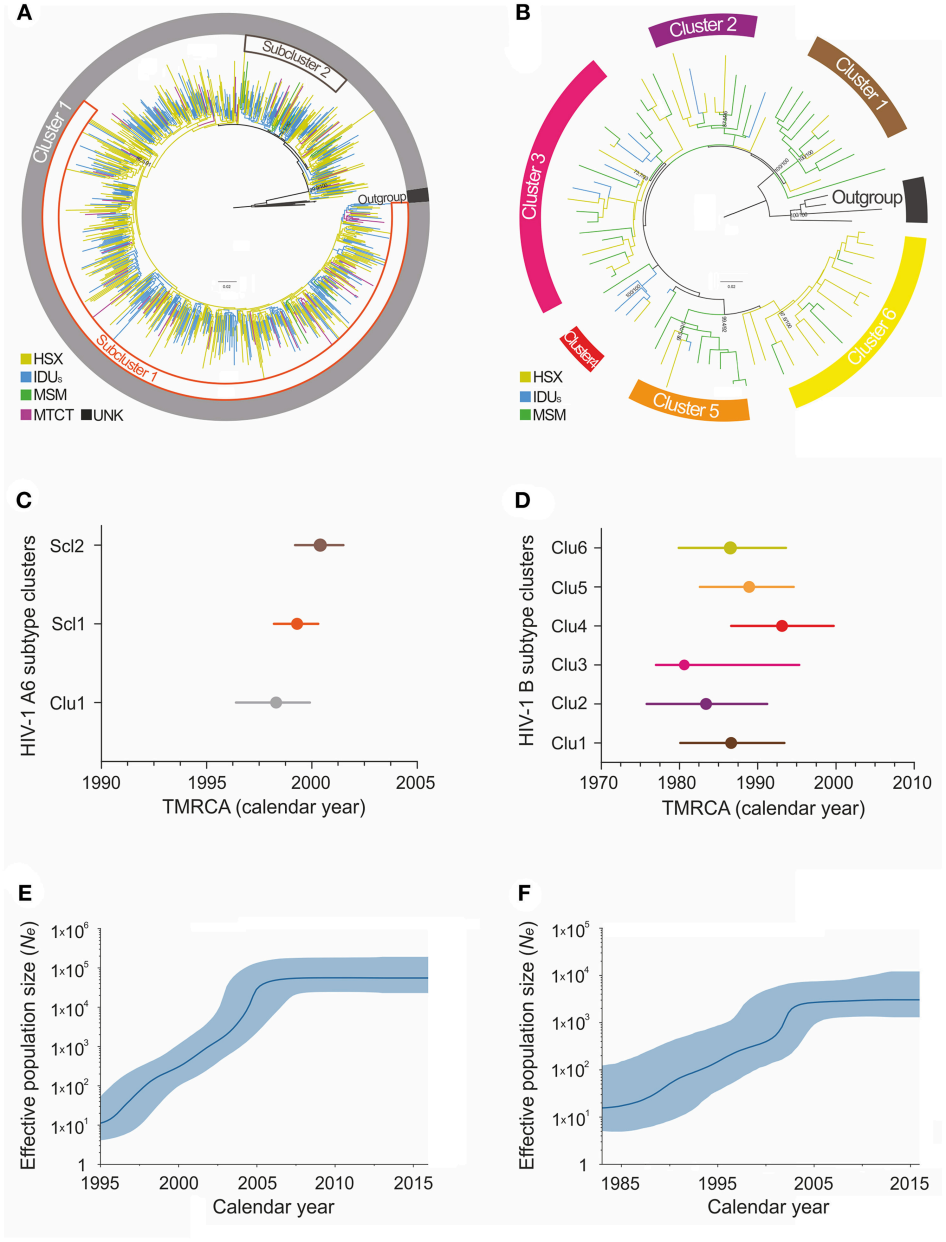
Maximum-likelihood phylogeny and phylodynamic analysis of HIV-1 subtypes in Moscow region. Shown is the analysis of subtypes A6 (left; **A,C,E**) and B (right; **B,D,F**). **(A,B)** ML-tree of the HIV-1 subtypes A and B pol sequences from HIV-infected persons in Moscow, 2007–2016. The colored areas indicate the positions of major epidemic clusters (sub-clusters) identified in the region. Branches are colored according to the risk factor of infection as indicated in the legends. Support values (BS/SH-aLRT) are shown for the key nodes. The trees were rooted through African A1 and D sequences (for A6 and B subtype, respectively). **(C,D)** Bayesian estimates of times of the most recent common ancestors (TMRCA) for the major epidemic clusters (sub-clusters) by BEAST analysis. Points represent the median value, solid lines - 95% credible intervals. **(E,F)** Bayesian skyline plot (BSP) showing effective number of infections over time Ne(t). Estimates of the Ne(t) are presented as median (solid blue line) with the corresponding 95% HPD credibility interval (blue area).

Maximum-likelihood and Bayesian phylogenetic analyses identified several clusters/subclusters of HIV-1 subtypes A6 and B (Figure 2 and Table 2). All 768 A6-sequences (100%) formed one cluster including sub-clusters (1-2). The proportion of sequences transmitted through HSX, IDU, MSM, and MTCT in these subclusters was comparable. The TMRCA [95% HPD] for Cluster 1 and subclusters 1 and 2 were estimated to be 1998.3 [1996.4-1999.9] - 2000.4 [1999.2-2001.5]. Analysis of subtype B sequences identified six clusters (1–6) including 64 (83.2%) sequences. Unlike A6, the genetic structuring of subtype B was to some extent dependent on the transmission route. The smallest cluster (cluster 4) contained ~5% of the B sequences obtained mainly from IDUs. While the sequences in cluster 3 (largest, ~25% sequences) were from IDUs and MSM-individuals (in equal proportion), they were predominantly from MSM in clusters 4 and 5, and from HSX-individuals in cluster 2 and 6. The TMRCA for clusters 1-6 were estimated to be 1980.6 [1977.4-1995.3] - 1993.1[1986.6-1999.7] (Figure 2 and Table 2).

**Table 2:** The cluster sizes, TMRCA, and transmission routes for major epidemic clusters of HIV-1 subtypes in Moscow region.

	**Sequence, *N* (%)**	**Transmission route**					***P*-value^**a**^**	**TMRCA [95% HPD]**
		**HSX, *N* (%)**	**IDUs, *N* (%)**	**MSM, *N* (%)**	**MTCT, *N* (%)**	**Unknown, *N* (%)**		
HIV-1 subtype A6							0.136^b^	
Cluster 1	768 (100)	389 (100)	323 (100)	6 (100)	44 (100)	6 (100)		1,998.3 [1,996.4–1,999.9]
Subcluster 1	380 (49.5)	205 (54.0)	144 (37.9)	2 (0.5)	24 (6.3)	5 (1.3)		1,999.3 [1,998.2–2,000.3]
Subcluster 2	90 (11.7)	41 (45.5)	45 (50.0)	0	4 (4.5)	0		2,000.4 [1,999.2–2,001.5]
Un(sub)cluster	298 (38.8)	143 (48.0)	134 (45.0)	4 (1.3)	16 (5.4)	1 (0.3)		–
All	768 (100)	389 (100)	323 (100)	6 (100)	44 (100)	6 (100)		–
HIV-1 subtype B							0.004^c^	
Cluster 1	9 (11.7)	2 (22.2)	0	7 (77.8)	–	–		1,986.6 [1,980.1–1,993.4]
Cluster 2	8 (10.4)	5 (62.5)	1 (12.5)	2 (25.0)	–	–		1,983.4 [1,975.8–1,991.2]
Cluster 3	19 (24.7)	5 (26.3)	4 (21.1)	10 (52.6)	–	–		1,980.6 [1,977.0–1,995.3]
Cluster 4	4 (5.2)	1 (25.0)	3 (75.0)	0	–	–		1,993.1 [1,986.6–1,999.7]
Cluster 5	9 (11.7)	2 (22.2)	1 (11.1)	6 (66.7)	–	–		1,988.9 [1,982.6–1,994.6]
Cluster 6	15 (19.5)	11 (73.4)	0	4 (26.6)	–	–		1,986.5 [1,979.9–1,993.6]
Uncluster	13 (16.8)	5 (38.5)	2 (15.4)	6 (46.1)	–	–		–
All	77 (100)	31 (40.3)	11 (14.3)	35 (45.4)	–	–		–

*HSX, Heterosexuals; MSM, men who have sex with men; IDUs, intravenous drug users; MTCT, mother-to-child transmission; TMRCA, time to the most recent common ancestor*.

a *Value for the difference between clusters (subclusters)*.

b *MSM and unknown transmission route were not considered in the analysis; Pearson's chi-squared test*.

c *Fisher's exact test (two-tailed)*.

### Demographic History and Evolutionary Rates of HIV-1 Subtypes A6 and B

According to the Bayesian analysis, the mean evolutionary rates [95% HPD] for subtypes A6 and B were 1.26 × 10^−3^ [1.13 × 10^−3^-1.41 × 10^−3^] substitutions/site/year and 1.17 × 10^−3^ [6.10 × 10^−4^ −1.82 × 10^−3^] substitutions/site/year, respectively. The Bayesian Skyline plot indicated at least two effective population growth phases for these subtypes (Figures 2E,F). While the population size of A6 grew exponentially between 1998 and 2004, reaching a stable phase (moderate growth) around 2006, subtype B had an initial phase of fast growth between 1985 and 2002 followed by a decline around 2003. Since the mid-2010s, both subtypes have shown a very slow increase.

### Recombinant Strains

We found 38 HIV-1 URFs containing genome segments of A6 and B subtypes. Of them, at least 12 (31.6%) had recombinant structures with similar breakpoint locations in the reverse transcriptase region. While the first fragment (2253 → 3342 ± 65) belonged to sub-subtype A6, the second fragment (3343 ± 65 → 3553) belonged to subtype B (Table 3).

**Table 3:** jpHMM-assigned breakpoint locations for 41 HIV-1 unique recombinant sequences in patients from Moscow region.

**Sequence name**	**Fragment (subtype)**			**Breakpoint**^**†**^	
	**1**	**2**	**3**	**1**	**2**
MOW177762	2,253–3,440 (CRF02)	3,454–3,553 (B)		3,447 ± 6	
MOW212318	2,253–2,322 (B)	2,454–3,553 (CRF02)		2,388 ± 65	
MOW105047	2,253–2,957 (B)	3,029–3,553 (CRF02)		2,993 ± 35	
MOW206415	2,253–2,544 (A6?/B)	2,552–3,352 (A6)	3,404–3,553 (B)	2,548 ± 3	3,378 ± 25
MOW212381	2,253–2,374 (B)	2,450–3,553 (A6)		2,412 ± 37	
MOW191857	2,253–3,397 (A6)	3,421–3,553 (B)		3,409 ± 11	
MOW179103	2,253–3,433 (A6)	3,461–3,553 (B)		3,447 ± 13	
MOW270119	2,253–2,735 (A6)	2,759–3,069 (B)	3,107–3,553 (A6)	2,747 ± 11	3,088 ± 18
MOW26246	2,253–2,312 (B)	2,402–3,553 (A6)		2,357 ± 44	
MOW102645	2,253–2,462 (B)	2,482–3,553 (A6)		2,472 ± 9	
MOW103145	2,253–2,375 (B)	2,517–3,553 (A6)		2,446 ± 70	
MOW109184	2,253–2,374 (B)	2,441–3,553 (A6)		2,408 ± 33	
MOW284149	2,253–2,699 (A6?/A1)	2,733–3,423 (A6)	3,433–3,553 (B)	2,716 ± 16	3,428 ± 4
MOW119218	2,253–3,272 (A6)	3,294–3,553 (B)		3,283 ± 10	
MOW126388	2,253–3,386 (A6)	3,406–3,553 (B)		3,396 ± 9	
MOW125543	2,253–2,474 (A6)	2,528–2,925 (B)	3,061–3,553 (A6)	2,501 ± 26	2,993 ± 67
MOW127873	2,253–3,452 (A6)	3,488–3,553 (B)		3,470 ± 17	
MOW170294	2,253–2,312 (B)	2,358–3,553 (A6)		2,336 ± 22	
MOW173915	2,253–2,397 (B)	2,457–3,553 (A6)		2,427 ± 29	
MOW129062	2,253–3,405 (A6)	3,422–3,491 (B)	3,501–3,553 (A6?/A4)	3,413 ± 8	3,496 ± 4
MOW178169	2,253–3,300 (A6)	3,398–3,553 (B)		3,349 ± 48	
MOW189742	2,253–3,340 (A6)	3,422–3,553 (B)		3,381± 40	
MOW230737	2,253–3,402 (A6)	3,422–3,553 (B)		3,412 ± 9	
MOW236022	2,253–3,295 (A6)	3,333–3,553 (B)		3,314 ± 18	
MOW243257	2,253–3,343 (A6)	3,399–3,553 (B)		3,371 ± 27	
MOW23690	2,253–3,327 (A6)	3,367–3,456 (B)	3,486–3,553 (A6)	3,347 ± 19	3,371 ± 14
MOW257204	2,253–2,362 (B)	2,440–3,553 (A6)		2,401 ± 38	
MOW259399	2,253–3,430 (A6)	3,440–3,553 (B)		3,435 ± 4	
MOW262312	2,253–2,312 (B)	2,374–3,553 (A6)		2,343 ± 30	
MOW263861	2,253–2,321 (B)	2,459–3,553 (A6)		2,390 ± 68	
MOW18862	2,253–2,786 (CRF02)	2,815–2,965 (A6)	3,011–3,553 (CRF02)	2,800 ± 14	2,988 ± 22
MOW269550	2,253–2,312 (B)	2,382–3,553 (A6)		2,347 ± 34	
MOW273948	2,253–3,304 (A6)	3,386–3,553 (B)		3,361 ± 24	
MOW283088	2,253–2,399 (B)	2,457–3,553 (A6)		2,428 ± 28	
MOW285258	2,253–2,666 (B)	2,734–3,553 (A6)		2,700 ± 33	
MOW302647	2,253–2,313 (B)	2,457–3,553 (A6)		2,385 ± 71	
MOW316504	2,253–2,948 (A6)	2,990–3,221 (B)	3,293–3,553 (A6)	2,969 ± 20	3,257 ± 35
MOW328606	2,253–3,342 (A6)	3,398–3,553 (B)		3,370± 27	
MOW243074	2,253–3,392 (A6)	3,422–3,553 (B)		3,407 ± 14	
MOW328087	2,253–3,406 (A6)	3,422–3,553 (A6)		3,414 ± 7	
MOW231027	2,253–3,307 (B)	3,401–3,471 (B)	3,489–3,553 (B)	3,354 ± 46	3,354 ± 8

† *Breakpoint locations (fragment coordinate) correspond to with HXB2 (K03455) numbering. Two HIV-1 sequences (MOW267311 and MOW262544) with multiple recombination points are not represented*.

## Discussion

This is the most extensive study in Moscow region to date, devoted to understanding the diversity and temporal dynamics of the common HIV-1 subtypes. We have confirmed that sub-subtype A6 (85.1%) is still the major HIV-1 subtype in the region (Giliazova et al., 2010) (as elsewhere in Russia) (Kazennova et al., 2011; Lapovok et al., 2017), followed by subtype B at 7.6%. The A6 subtype predominates in HSXs and IDUs while subtype B is the most prevalent variant in MSM group. This unequal distribution repeats the molecular pattern of the epidemic in Russia as a whole. Apparently, the ≪ founder effect≫ and the relative social isolation/persistence of individual risk groups (at least at the beginning of the epidemic) are responsible for this phenomenon. We also observed a significant spread (4.2%) of unique recombinants between the two main subtypes, with at least 11 sequences distinct from the currently described A6/B recombinants. We are planning further studies with these recombinants, including full-length genome analysis to determine if they are new circulating forms. The CRF02_AG is the fourth most common HIV variant in the region, which is not surprising given the high level of labor migration from Central Asia where this subtype is widespread. Finally, we have identified at least seven other HIV-1 subtypes, which points to the increasing genetic complexity of the HIV-1 epidemic in the region.

We also investigated the emergence of HIV-1 A6 and B epidemics and their growth rates in Moscow region using the molecular clock. According to our estimates, A6 sub-subtype was introduced into the region around 1998 and marked the onset of massive epidemic (Figure 2). These findings are in good agreement with other sero-epidemiological studies (Bobkov et al., 2001). Subsequently, the divergent evolution of A6 strains and possible existence of several independent transmission networks apparently led to the formation of two epidemic sub-clusters, identified in this study.

The study of the genetic relationships between HIV-1 B-strains revealed that the spread of the subtype B in Moscow region involved at least six viral lineages that arose between 1980 and 1993 (Figure 2). Clusters 1-3, 5, and 6, which also belong to the Pandemic subtype B were responsible for 70.1% of the sexually transmitted infections and played a dominant role in the B-epidemic in Moscow region among MSM and HSXs. On the other hand, the FSU-B strain (Cluster 4) was responsible for infections in IDUs.

Though we are confident that HIV-1 subtype B penetrated into the region much earlier than A6, the origin of these clusters deserves further investigation. The growth pattern of the B epidemic is similar to that of A6 and represents the combined population dynamics of different subtype B-clusters. With an initial phase of rapid growth (not as rapid as for subtype A6), it appears that subtype B encountered less favorable conditions for local expansion, such as a slower rate of sexual transmission (Maljkovic Berry et al., 2007). The implementation of ART among all population groups could account for the slowdown in the epidemic since the mid-2010s.

The study can contribute to a better understanding and assessment of the social and biological driving forces behind the development of the epidemic process in one of the key regions for the HIV epidemic in Russia, as well as predicting of future trends of HIV infections and suggesting effective preventive measures.

## GenBank Accession Numbers

All HIV-1 sequences described in this study were submitted to Genbank (accession numbers: MH666355-667255; KY857892-857922).

## Ethics Statement

This study was approved by the local Ethics Committee of the Moscow Regional AIDS Centre (RCAC). Informed consent was signed by all the subjects.

## Author Contributions

AL and MB planned and designed the study. NL and AP collected the epidemiological data and nucleotide sequences. FM performed the recombination analysis and submitted sequences. EK performed the analysis of the epidemiological data. AL performed the phylogenetic and phylodynamic analysis, produced the illustrations and wrote the manuscript. MB supervised the project and edited the manuscript. All authors participated in the critical review of this manuscript.

### Conflict of Interest Statement

The authors declare that the research was conducted in the absence of any commercial or financial relationships that could be construed as a potential conflict of interest. The reviewer DP declared a past co-authorship with one of the authors MB to the handling editor.
