# Supplementary material for: Human Immunodeficiency Virus-1 Diversity in the Moscow Region, Russia: Phylodynamics of the Most Common Subtypes
Source: Front Microbiol. 2019 Feb 26;10:320. doi: 10.3389/fmicb.2019.00320 (PMC6399469; doi:10.3389/fmicb.2019.00320)
Supplement: Supplementary file 1 [file Data_Sheet_1.docx]

**
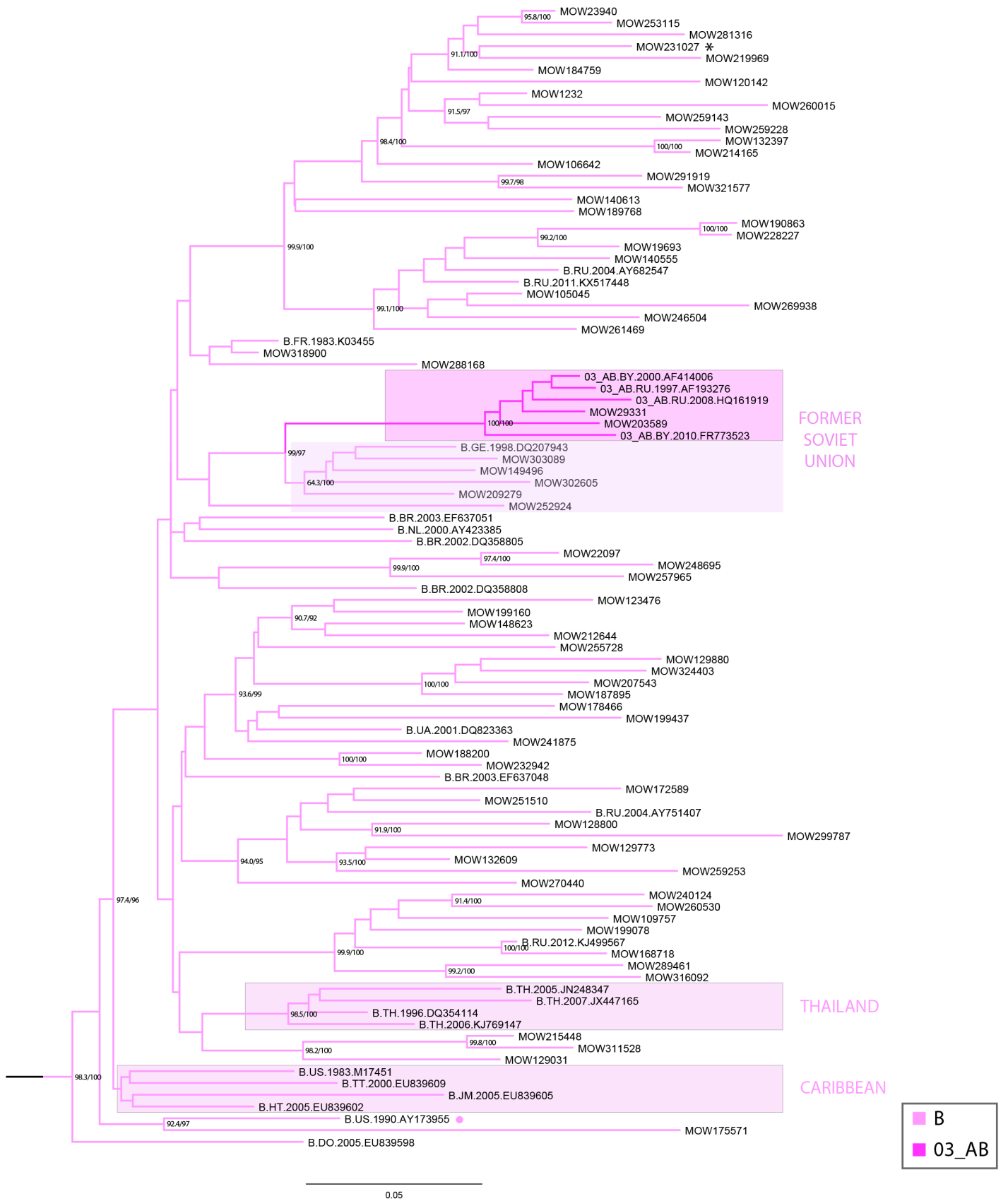
**

**Supplemental Figure S1**. The topology of the A6-clade and B/03_AB-clade for maximum-likelihood phylogenetic tree of 896 HIV-1 pol sequences from HIV-infected individuals in Moscow, from 2011-2016 (see main text). Branches are colored according to the HIV-1 subtypes as indicated in the legend. Support values (BS/SH-aLRT) are shown for the key nodes. Asterisks point to locations of unique recombination forms. The reference sequence for each subtype was selected using the Reference Alignment from Rega HIV-1 Subtyping Tool and marked by a circle.

**
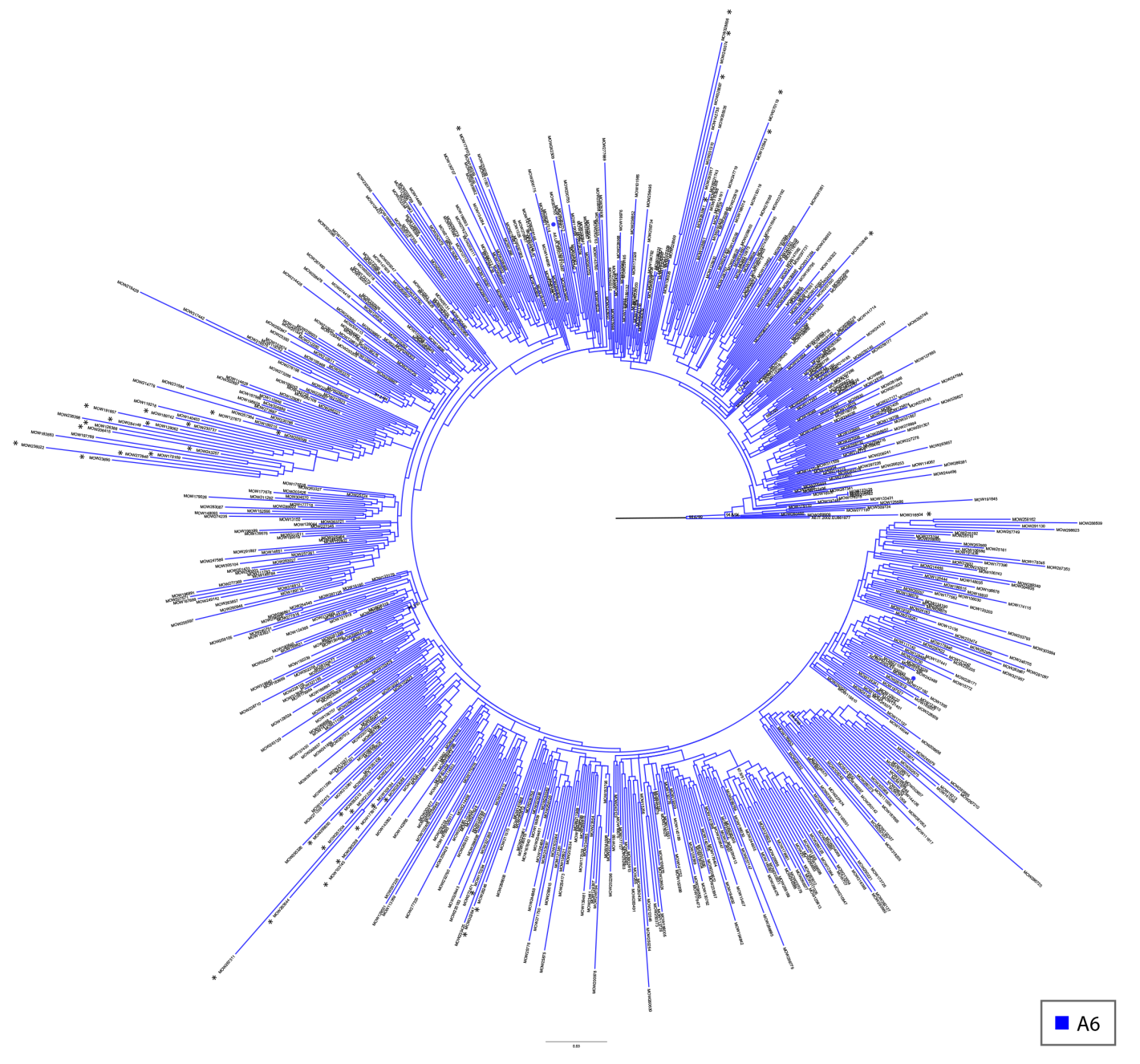
**

**Supplemental Figure S1**. Continued.
